# Phytochemical Compounds as Promising Therapeutics for Intestinal Fibrosis in Inflammatory Bowel Disease: A Critical Review

**DOI:** 10.3390/nu16213633

**Published:** 2024-10-25

**Authors:** Aya A. Touny, Balaji Venkataraman, Shreesh Ojha, Mauro Pessia, Veedamali S. Subramanian, Shamanth Neralagundi Hariharagowdru, Sandeep B. Subramanya

**Affiliations:** 1 Department of Physiology, College of Medicine and Health Sciences, United Arab Emirates University, Al Ain P.O. Box 15551, United Arab Emirates; aya.abdelraouf@acu.edu.eg (A.A.T.); balajiv@uaeu.ac.ae (B.V.); mauro@uaeu.ac.ae (M.P.); shamanth@uaeu.ac.ae (S.N.H.); 2 Department of Clinical Pharmacy and Pharmacy Practice, Faculty of Pharmacy, Ahram Canadian University, Giza 12581, Egypt; 3 Department of Pharmacology and Therapeutics, College of Medicine and Health Sciences, United Arab Emirates University, Al Ain P.O. Box 15551, United Arab Emirates; shreeshojha@uaeu.ac.ae; 4 Department of Medicine, University of California, Irvine, CA 92697, USA; vsubrama@uci.edu; 5 Zayed Bin Sultan Center for Health Sciences, College of Medicine and Health Sciences, United Arab Emirates University, Al Ain P.O. Box 15551, United Arab Emirates

**Keywords:** intestinal fibrosis, IBD, TGF-beta, phytochemicals and health, polyphenols

## Abstract

Background/Objective: Intestinal fibrosis, a prominent consequence of inflammatory bowel disease (IBD), presents considerable difficulty owing to the absence of licensed antifibrotic therapies. This review assesses the therapeutic potential of phytochemicals as alternate methods for controlling intestinal fibrosis. Phytochemicals, bioactive molecules originating from plants, exhibit potential antifibrotic, anti-inflammatory, and antioxidant activities, targeting pathways associated with inflammation and fibrosis. Compounds such as Asperuloside, Berberine, and olive phenols have demonstrated potential in preclinical models by regulating critical signaling pathways, including TGF-β/Smad and NFκB, which are integral to advancing fibrosis. Results: The main findings suggest that these phytochemicals significantly reduce fibrotic markers, collagen deposition, and inflammation in various experimental models of IBD. These phytochemicals may function as supplementary medicines to standard treatments, perhaps enhancing patient outcomes while mitigating the adverse effects of prolonged immunosuppressive usage. Nonetheless, additional clinical trials are necessary to validate their safety, effectiveness, and bioavailability in human subjects. Conclusions: Therefore, investigating phytochemicals may lead to crucial advances in the formulation of innovative treatment approaches for fibrosis associated with IBD, offering a promising avenue for future therapeutic development.

## 1. Introduction

Intestinal fibrosis is a chronic and progressive disease that starts as a complication of the persistent and long-lasting inflammation associated with inflammatory bowel disease (IBD). It affects more than one-third of IBD patients within ten years of disease onset, and it accelerates morbidity and mortality, resulting in the need for hospitalization and surgery [1]. Intestinal fibrosis is considered a multifactorial disease caused by a cascade of events due to the interplay between molecular and cellular mechanisms that induce the process of fibrogenesis, including pro-inflammatory and profibrotic cytokines, particularly the profibrotic protein transforming growth factor β (TGF-β), gut microbiota, and mesenchymal cells, especially fibroblasts and myofibroblasts [2,3]. Although the pathophysiology behind intestinal fibrosis is complex, the underlying cause of the progression of IBD into fibrosis is hypothesized to be a protracted, unresolved chronic inflammation that causes a defective repair due to persistent intestinal tissue injury [4,5]. To repair the recurrent mucosal damage, extracellular matrix (ECM) is produced extensively; therefore, intestinal fibrosis is characterized by fibrotic strictures of ECM, particularly collagen, which is accumulated and deposited transmurally, resulting in a narrowing of the lumen [6].

In ulcerative colitis (UC), the mucosal and submucosal layers of the colon are the only locations where ECM is deposited. In contrast, in Crohn’s disease (CD), the layers of mucosa, submucosa, muscularis mucosa, muscularis propria, and serosa of the whole intestinal wall can be affected by fibrosis [7].

TGF-β is a pleiotropic protein that plays physiological and pathological roles. Physiologically, it is crucial for immune response regulation, tissue injury healing, and the growth, proliferation, differentiation, and migration of cells [8,9]. Dysregulated TGF-β signaling has been identified as the key force of all signaling mechanisms and aberrant pathological activities that drive fibrogenesis [10]. The overexpression of the TGF-β1/Smad signaling pathway is the underlying trigger of intestinal fibrosis, as several studies have proven [4,11,12]. On the other hand, research findings indicate that the downregulation of this TGF-β1/Smad pathway limits the activation of the human intestinal fibroblasts which serve as the precursor of myofibroblasts; the major collagen-producing and alpha-smooth muscle actin (α-SMA) expressing cells. Thus, this downregulation results in the suppression of α-SMA and ECM deposition via inhibition of the phosphorylated Smad2, -3, and -4 expressions while increasing that of Smad7, which serves as a negative regulator of TGF-β [13,14].

No therapies have been approved for intestinal fibrosis prevention or treatment that adequately meet the high clinical demand. Anti-inflammatory and immunosuppressive medications, the standard of care for IBD, are administrated to attenuate the causative inflammation and delay the fibrogenesis process. However, they do not relieve or reverse fibrosis, making surgical intervention inevitable in IBD patients [15,16]. In the meantime, more strictures can emerge in other locations of the bowel, ultimately leading to further surgical intervention [17]. Therefore, further drug development expansion and different therapeutic approaches are essential.

Medicinal plant products have been administered for thousands of years to treat different diseases. They have fewer adverse effects, better availability, and are affordable. They act as lead compounds with various biologically active ingredients, which can be new drug candidates [18,19]. Interestingly, several phytochemical compounds, such as calycosin, asperuloside and many others, have exhibited efficacy in managing multiple health conditions, including IBD; therefore, they could also fill in the gaps in the treatment of IBD-associated fibrosis [20,21,22].

This review aims to summarize the possible mechanisms of intestinal fibrosis pathogenesis, emphasizing the master role of TGF-β, the current therapeutic approaches, and the promising phytochemical drug candidates.

## 2. Methodology

Electronic databases, including PubMed, Scopus, Google, and Google Scholar, were searched using keywords such as “intestinal fibrosis”, “TGFβ”, “SMAD”, “microbiome”, “phytochemical”, “plant”, “natural product”, “plant-based nutraceuticals”, “bioactive molecules”, and “therapies”. This review focuses on studies conducted between 1987 and 2024 using in vivo and in vitro experimental models of intestinal fibrosis. It also emphasizes the potential benefits of phytochemicals and plant-based medicines for treating intestinal fibrosis. The sources of phytochemicals, experimental models, and reported effects/mechanisms are summarized in tables.

## 3. Pathophysiology of Intestinal Fibrosis (Cellular and Molecular Insight)

Several cellular and molecular pathways have been demonstrated to play an intricate role in regulating the cell signaling pathways and contributing to the development of fibrosis.

### 3.1. The Interaction Between TGF-β1 and the Other Pathways

The most recognized driver of fibrosis in both intestinal and extraintestinal organs is the versatile cytokine TGF-β, particularly its TGF-β1 isoform [4,23,24]. TGF-β1 directly activates Smad-dependent and Smad-independent signaling pathways to initiate fibrogenesis.

#### 3.1.1. TGF-β1 and Smad Pathway

**Figure 1:**
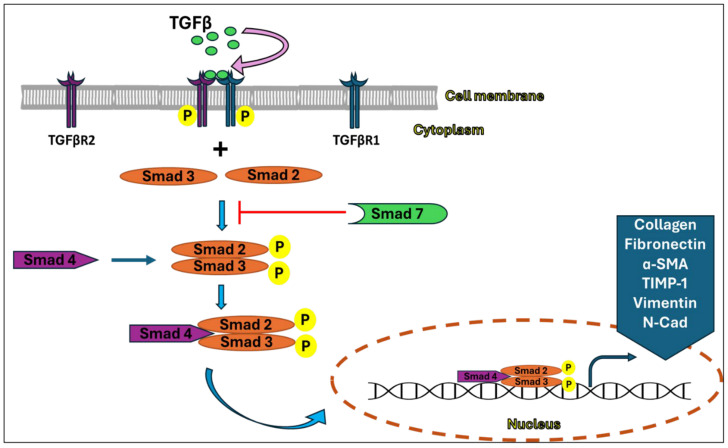
Activation of the components of the TGFβ/Smad canonical signaling pathway initiates the fibrosis process by inducing transcription of the downstream fibrotic genes. Abbreviations: α-SMA—alpha-smooth muscle actin, TIMP—tissue inhibitors metalloproteinases, N-Cad—Neural cadherin.

TGF-β1 triggers intracellular signals and phosphorylation of Smad2 and Smad3 proteins when it binds to its receptors, TGFβ-receptor 1 (TGFβ-R1) and TGFβ-receptor 2 (TGFβ-R2). These signals are further amplified and spread, however, they can be antagonized by Smad6 or Smad7 which counter-regulate the TGFβ signaling by impeding the ligation of Smad 2/3 to the active receptor complex [25,26]. Afterwards, the phosphorylated Smad2 and Smad3 form a complex with Smad4, followed by the translocation of this complex into the nucleus, in turn inducing the expression of the target downstream fibrotic genes, most importantly α-SMA, collagen, connective tissue growth factor (CTGF) and fibronectin (Figure 1) [27,28].

#### 3.1.2. TGF-β1 and NFκB Pathway

The activation of nuclear factor kappa B (NFκB) signaling is triggered by multiple stimuli, including cytokines such as tumor necrosis factor-α (TNF-α), interleukin-1β (IL-1β), and TGF-β. TGF-β can stimulate NFκB through both the canonical Smad and the non-canonical Smad pathways. Smad3 can interact directly with the NFκB basic proteins. However, TGF-β can stimulate NFκB through transforming growth factor-β-activated kinase-1 (TAK1) in the non-canonical Smad pathway. On the other hand, a study has revealed that TGF-β promotes the overexpression of IL-1β and TNF-α cytokines, which in turn activates the transcription of NFκB and its target genes. Furthermore, it has been shown that TGF-β/NFκB signaling pathway activation promotes collagen gene expression and epithelial–mesenchymal transition (EMT) [29,30].

#### 3.1.3. TGF-β and MAPK Pathway

**Figure 2:**
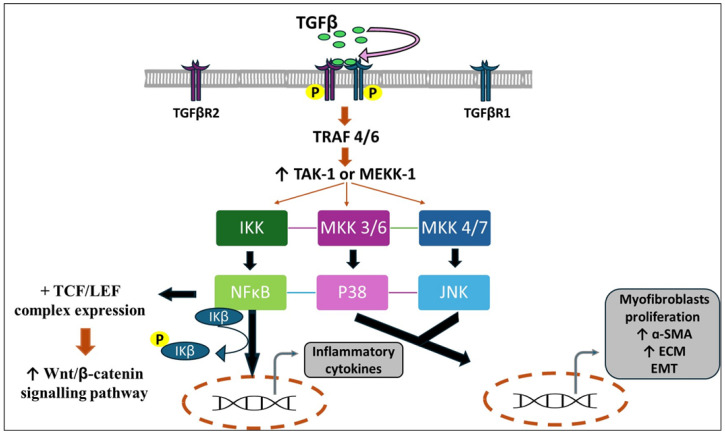
Activation of TGF-β stimulates NFκB and MAPK signaling pathways with the subsequent activation of the Wnt/β-catenin pathway. Abbreviations: TRAF—TNF receptor associated factor, TAK1—transforming growth factor-β activated kinase 1, MEKK1—mitogen-activated protein kinase kinase1, IKK—inhibitor of nuclear factor-κB (IκB) kinase, MKK—mitogen-activated protein kinase kinase, IKβ—I-kappaB kinase, ECM—extracellular matrix, EMT—epithelial–mesenchymal transition (↑—increased).

Through the Smad independent pathway, TGF-β activates the mitogen-activated protein kinase (MAPK), and the downstream factors, including extracellular signal-regulated kinases (ERK), p38, c-Jun *N*-terminal kinases (JNK) kinases, and NFκB which are driven by MEK 1/2, MKK 3/6, MKK 4/7 and IKK, respectively. The cascade of these intracellular signaling molecules induces EMT, myofibroblast formation, and ECM buildup (Figure 2) [31,32,33].

#### 3.1.4. TGF-β1 and TAK 1

TAK1, which is part of the MAPK kinase family mediates the TGF-β activation of the p38 MAPK pathway. The p38 MAPK signaling is responsible for the collagen gene expression triggered by TGF-β [34,35].

#### 3.1.5. TGF-β1 and Wnt/β-Catenin Pathway

There is crosstalk between the wingless-related integration site (Wnt) and the TGF-β-Smad pathways that drives the fibrogenesis process [36]. NFκB signaling is a central mediator of inflammation, and its dysregulation is associated with a wide range of inflammatory responses [37,38,39,40]. Many studies have demonstrated an interaction between the NFκB signaling and the Wnt/β-catenin pathways, whereas this interaction modulates immune and inflammatory responses [41]. The canonical Wnt/β-catenin pathway can be activated by NFκB signaling, which results in the stimulation of TCF/LEF complex expression, which is the pivotal downstream mediator of the Wnt signaling, and hence the upregulation of the Wnt/β-catenin pathway [42,43,44,45,46,47]. Furthermore, the inflammatory reactions are triggered by the activation of β-catenin target genes mediated by Wnt/β-catenin pathway overexpression [48,49].

**Figure 3:**
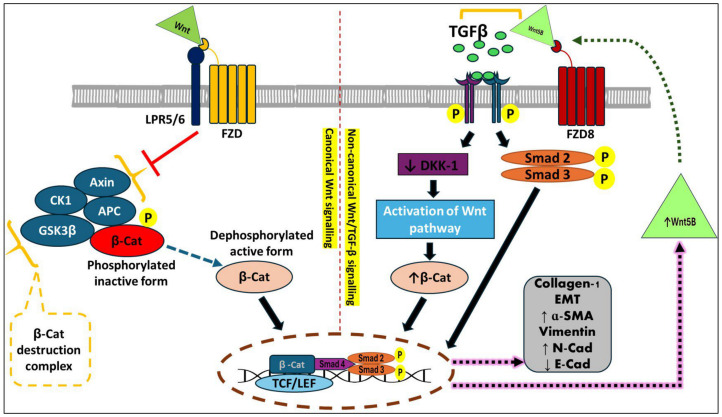
Activation and interaction of both canonical Wnt signaling and non-canonical Wnt/TGF-β. Abbreviations: LPR5/6—low density lipoprotein receptor-related proteins 5/6, FZD—seven-pass transmembrane receptor frizzled, CK1—casein kinase 1, APC—adenomatosis polyposis coli, GSK3β—glycogen synthase kinase 3β, DKK1—Dickkopf WNT signaling pathway inhibitor1, β-Cat—β-catenin, TCF/LEF—T cell factor/lymphoid enhancer factor, N-Cad—neural cadherin, E-Cad—Epithelial cadherin (↑—increased; ↓—decreased).

On the other hand, previous studies have indicated that TGF-β modulates Wnt signaling, which can be both β-catenin-dependent and independent when inducing fibrosis, proving that intestinal fibrotic tissues have elevated levels of β-catenin [50,51]. TGF-β triggers noncanonical Wnt/TGF-β signaling, which is mediated by Wnt5B and FZD8, which in turn are the Wnt signaling pathway components that are elevated in intestinal strictures. On the other hand, TGF-β can directly upregulate FZD8, which in turn facilitates the TGF-β-induced collagen deposition [36]. Activation of the non-canonical Wnt/TGF-β pathway promotes Wnt pathway stimulation by inhibiting the dickkopf-related protein (DKK), which is a primary suppressor of the Wnt pathway, resulting in the increase of β-catenin levels followed by the transcription of downstream fibrotic genes [52,53]. Meanwhile, Wnt ligands bind to seven-pass transmembrane receptor frizzled (FZD) receptors, resulting in the canonical Wnt signaling activation, including suppression of the β-catenin destruction complex and elevation of the dephosphorylated active form of β-catenin. Then, β-catenin is translocated into the nucleus, leading to the activation of T cell factor/lymphoid enhancer factor (TCF/LEF) TCF/LEF-dependent gene transcription and upregulation of collagen-I expression (Figure 3) [36].

**Figure 4:**
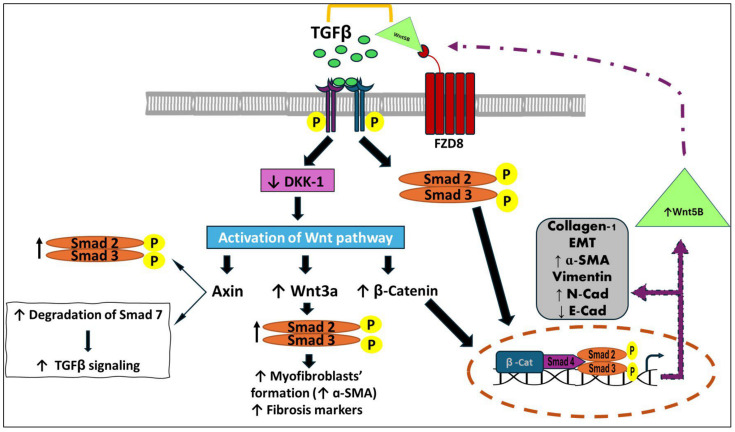
Interaction between non-canonical Wnt/β-catenin and TGFβ/Smad signaling pathways to stimulate the fibrogenesis process and transcription of the downstream fibrotic genes. Abbreviations: DKK1—Dickkopf WNT signaling pathway inhibitor 1, β-Cat—β-catenin, EMT—epithelial–mesenchymal transition, N-Cad—neural cadherin, E-Cad—epithelial cadherin. (↑—increased; ↓—decreased).

When TGF-β and Wnt are stimulated, axis inhibition protein (Axin) induces Smad 2/3 binding to the TGFβ-R1 and allows its phosphorylation [54]. Then, the canonical Wnt ligands, particularly Wnt3a, activate Smad2 in a β-catenin-dependent mechanism to promote TGF-β1 expression and Smad2/3 phosphorylation, resulting in EMT and the transformation of fibroblasts into myofibroblasts [55,56]. Additionally, Axin boosts Smad7 degradation in response to Wnt activation and, as previously indicated, Smad7 is essential to antagonize the TGF-β signaling (Figure 4) [57].

#### 3.1.6. TGF-β and PAI-1

Plasminogen activator inhibitor-1 (PAI-1) is regulated by TGF-β and is one of its downstream targets during signal transmission [58]. Under normal physiological conditions, PAI-1 acts in a manner that controls fibrinolysis to maintain a balance between ECM production and degradation. It inhibits the tissue plasminogen activator (tPA) which catalyzes the conversion of plasminogen into plasmin, leading to the activation of the matrix metalloproteinases (MMPs) which degrade the ECM, preventing its accumulation [59,60].

On the other hand, it has been reported that PAI-1 is overexpressed in the inflamed lesions and fibrotic tissues of IBD patients [61]. During chronic inflammation, TGF-β upregulates the levels of PAI-1 via Smad protein activation, resulting in the inactivation of tPA which in turn blocks the enzymatic conversion of plasminogen to plasmin. Consequently, the suppression of matrix metalloproteinases-9 (MMP-9) occurs leading to a dramatic inhibition in the ECM degradation, and hence accumulation of collagen [62,63]. These findings suggest that PAI-1 is a promising and potential therapeutic target in the treatment of fibrosis [63].

### 3.2. AXL

The AXL (from the Greek “anexelekto”, meaning uncontrolled) is one of the tyrosine kinase receptors known as TAM (TYRO 3, AXL, and MER). AXL signaling contributes to the endo–MT, and is involved in the activation of the myofibroblasts [64,65]. It has been found that TGF-β and its signaling mediate expression of AXL and promote liver and kidney fibrosis. A study has investigated the implication of AXL in intestinal fibrosis and demonstrated that the blocking of AXL expression by a specific inhibitor known as BGB324 leads to the downregulation of the fibrogenic genes. Additionally, the inhibition of AXL was found to be associated with the induction of fibroblast apoptosis by Fas ligand (FasL). Thus, developing antifibrotic agents targeting AXL is promising for preventing and treating fibrosis [66].

### 3.3. MMPs and TIMPs

The level of ECM is controlled by two groups of enzymes or proteins known as the matrix metalloproteinases (MMPs) and the tissue inhibitors metalloproteinases (TIMPs). MMPs maintain the level of ECM by balancing between ECM degradation and formation. Meanwhile, MMP is regulated and suppressed by TIMP to maintain that balance [67,68,69]. The dysregulation of the equilibrium between MMPs and TIMPs leads to incessant collagen deposition and the progression into fibrosis [70].

### 3.4. Cells Involved in the Initiation and Progression of Intestinal Fibrosis

#### 3.4.1. Mesenchymal Cells, Fibroblasts and Myofibroblasts

Mesenchymal cells, including fibroblasts and myofibroblasts, are the key cells implicated in the onset of the fibrosis process. During normal tissue repair response, inflammation triggers a series of events involving mesenchymal cell activation, particularly intestinal myofibroblasts, for wound healing and tissue repair mediated by these myofibroblasts to restore normal physiological homeostasis. This response is strictly controlled, limiting the myofibroblasts’ migration, proliferation, and ECM formation, followed by the inflammation resolution [68,69]. However, during the pathological tissue remodeling that is due to sustained inflammation and colonic tissue injury, failure of tissue healing occurs, which results in excessive ECM deposition, particularly collagen I and collagen III; thickening of the intestinal wall; bowel stricture; and obstruction [71,72]. Over-synthesis and production of ECM are driven by the differentiation of fibroblasts into myofibroblasts, with an increase in the expression of α-SMA, the myofibroblasts’ specific biomarker. Afterwards, the α-SMA-expressing myofibroblasts migrate and expand in number, accumulating more ECM [70,72,73,74]. Meanwhile, chronic inflammation and the released cytokines induce EMT and endothelial–mesenchymal transition (endo–MT). Upon initiating the fibrosis process, these epithelial and endothelial cells lose their characteristics and functions and acquire the fibroblasts’ phenotype [75,76].

Moreover, stimulation by cytokines or growth factors, particularly by TGF-β, activates fibroblasts which become more proliferative and migratory and differentiate rapidly into myofibroblasts which overexpress the α-SMA, indicating a dramatic differentiation of fibroblasts into myofibroblasts. Hence, myofibroblasts resist apoptosis and start a rapid proliferation, migration, and expansion [77]. Myofibroblasts are the major source of ECM, and they start to expand in number, driving the accumulation of ECM proteins, especially collagen, and resulting in an increase in the thickness of the intestinal wall and tissue fibrogenesis [70,72,73]. Myofibroblasts’ phenotype is manifested by the expression of α-SMA marker, which is usually measured and quantified to reflect the presence of myofibroblasts. The higher expression of α-SMA indicates that more myofibroblasts are being produced [78].

#### 3.4.2. Immune Cells

Intestinal tissue injury is induced by environmental factors and antigens that cross the intestinal epithelium, activating the antigen-presenting cells and driving the transformation of naïve T cells to natural killer T cells, Th1, Th2, and Th7, which release different proinflammatory cytokines. Th2 cells, for example, release IL-4, IL-5, and IL-13. MMP production can be inhibited by IL-13, leading to increased ECM deposition and elevated TGF-β activity [79].

The prolonged inflammation and infiltration of immune cells damage the mucosal architecture, which aggravates tissue injury and promotes the fibrogenesis process [80]. In addition, chronic inflammation and the release of inflammatory mediators that is promoted by continuous epithelial and endothelial damage boost the activation of cells producing ECM, particularly fibroblasts and myofibroblasts [68,81]. On the other hand, immune cells maintain myofibroblasts’ continual activation and proliferation by the secretion of more cytokines [82].

### 3.5. Role of Microbiota in Intestinal Fibrosis

Inflammation and fibrosis can result from host–microbiome interplays, which are disrupted by changes in the intestinal barrier function, gut microbiota, or immune system [83]. Most studies that have revealed the pathophysiological mechanisms of IBD have reported that disruption of the gut microbiota is a driving factor of those mechanisms [84,85,86]. Gut microbiota contributes to the pathological process of fibrosis by promoting adhesion, migration, and differentiation of fibroblasts into myofibroblasts [87]. For instance, gut bacteria known as adherent-invasive *Escherichia coli* (AIEC) have been linked to IBD, particularly CD [88,89]. According to one research study, acute inflammation is accompanied by AIEC intestinal colonization, resulting in fibrosis via the upregulation of ST2 expression, the IL-33 receptor, with the help of flagellin [90]. Furthermore, fibrosis is associated with the existence of *Salmonella enterica*, *Streptococcus*, *Lactobacillus*, *Mucispirillum schaedleri*, and *Ruminococcus* in the cecum as well as ileum [87,91].

In addition, inflammation and dysbiosis disrupt the integrity of the intestinal epithelial barrier, which allows the exposure of gut microbiota to immune and mesenchymal cells [92]. As a result, toll-like receptors (TLR), particularly TLR-4, that are expressed by the intestinal immune and non-immune cells worsen inflammation and activate signals that promote collagen deposition and fibrosis [93]. Additionally, two decades ago, a study reported that injection of the rat colonic wall with bacteria from the gut flora stimulated TGF-β1 release and collagen deposition [94].

Lipopolysaccharide (LPS) is a component of the outer membrane of gram-negative bacteria that was found to be fibrogenic. Upon exposure of fibroblasts to LPS, the TLR-4 located on the fibroblasts’ membrane recognizes LPS and recruits MyD88, an adaptor protein for TLR signaling, to its toll-interleukin-1 receptor, leading to the phosphorylation and activation of NF-κB [95,96,97]. Ultimately, this results in the suppression of Smad7, the TGF-β1 negative regulator, allowing the overexpression of TGF-β1 and the subsequent increase of ECM-producing collagen [98,99]. On the other hand, direct exposure of fibrocytes to LPS can induce the development of fibrosis independently of TGF-β1 stimulation [100].

Through similar mechanisms, a study found that peptidoglycan, a polysaccharide in the bacterial cell wall, can upregulate TGF-β1 and induce collagen overproduction by stimulating myofibroblasts [101].

In contrast, according to several studies, the administration of oral probiotics, such as lactic acid bacteria, also known as LAB, and Bifidobacterium species, may promote the restoration of the gut microbiome’s composition and preserve the integrity of the intestinal mucosa [102,103,104,105]. Several *Lactiplantibacillus plantarum*, which are strains of LAB, have been demonstrated to have therapeutic effects on animal models as well as in patients with IBD, particularly colitis [106,107,108,109,110].

### 3.6. Role of microRNAs in Intestinal Fibrosis

MicroRNAs (miRNAs) are a group of non-coding RNAs consisting of 18–25 small nucleotides that are responsible for the regulation of gene expression by degrading mRNA or repressing the translation [111,112,113]. Aberrant expression of miRNAs contributes to the pathogenesis of multiple pathological conditions in cancer, inflammation, and autoimmune diseases [114,115,116].

Several research outcomes have demonstrated that dysregulated miRNAs are implicated in colitis-associated fibrosis [2]. For example, when pre-miRNA-29b was transfected into intestinal fibroblasts, it induced the elevation of mRNA and protein expression of IL-6 and IL-8. Furthermore, it promotes the upregulation of collagen in the mucosa of CD patients [117]. MiRNA-155 is upregulated by TGF-β1 [118]. It has proven to display proinflammatory activity by increasing the levels of TNF-α, IL-6, IL-1β, and CCL2 cytokines [119]. Furthermore, another study has shown that miRNA-155 levels are elevated in the fibroblasts, where it promotes ECM deposition [120]. An interplay between miRNA-155 and the Wnt/β-catenin pathway has been revealed [121]. Under homeostatic conditions, activation of the Wnt signaling pathway by phosphorylation of GSK3β results in the accumulation of β-catenin, which promotes cells’ proliferation and collagen production [122]. Meanwhile, this process is controlled by the HBP-1 gene, which is a negative regulator of the Wnt/β-catenin pathway, through the suppression of the TCF–β-catenin complex which eventually allows control over the level of collagen production so as to prevent the development of fibrosis. However, when the human colonic CCD-18Co myofibroblast cells were transfected with miR-155, HBP-1 expression was significantly attenuated. Accordingly, the HBP-1 gene has been reported to be the direct target of miR-155. Therefore, overexpression of miRNA-155 downregulates the expression of HBP-1, leading to upregulation of the Wnt/β-catenin pathway, including its genes, such as phosphorylated GSK3β, TCF4, LEF, LGR5, and Myc, and followed by overexpression of the fibrosis markers α-SMA and collagen I, III and IV. To this end, the activation of the Wnt/β-catenin signaling pathway is associated with miRNA155-induced intestinal fibrosis [123].

Likewise, miRNA-21 is considered a profibrogenic molecule. TGF-β plays an important role in driving the maturation and the action of miR-21. When the TGF-β/Smad signaling pathway is activated, the translocation of the p-Smad2/3 and Smad4 complex into the nucleus promotes the pri-miR-21 conversion into pre-miRNA-21, which is then released as mature miR-21. This miR-21 suppresses Smad7, resulting in the TGF-β/Smad signaling pathway activation that initiates the fibrogenesis process [124]. Additionally, the upregulation of miR-21 results in the downregulation of PTEN, which serves as a negative regulator of the PI3K/AKT/mTOR pathway. Consequently, the mTOR pathway will be activated uncontrollably so as to enhance the EMT process and fibrosis [125].

MiR-130 contributes to the fibrosis process by the activation of the TGF-β/Smad pathway [126]. Similarly, miR-132 plays an important role in promoting myofibroblasts’ proliferation and collagen accumulation [127].

Other miRNAs, including miR-27, miR-29, and miR-30, show antifibrotic activities by inhibiting TGF-β/Smad signaling and the downstream fibrosis genes. Additionally, they attenuate the EMT and the ECM deposition [128,129,130,131,132].

## 4. Diagnosis

Currently, no accurate or definite biomarkers and imaging methods are available to quantitatively determine the degree of fibrosis. However, clinical studies rely only on the ECM alterations as the clinical endpoints [133].

The mainstay for diagnosing gastrointestinal conditions marked by mucosal changes is the gastrointestinal endoscopy or ileocolonoscopy. Stenosis is a constriction of the intestinal lumen due to the deposition of fibrous tissue, which prevents the endoscope from passing through. Thus, its role in assessing the extent of the stricture and the fibrosis components is restricted. Hence, biopsy samples can be collected for histological analysis and exclusion of malignancies [134,135].

Diagnosis of intestinal fibrosis can be clinically achieved after strictures are formed. Stenoses can be identified using a variety of cross-sectional imaging methods as alternatives to ileocolonoscopy, such as magnetic resonance imaging (MRI), computer tomography enterography (CTE), and ultrasound (US). These cross-sectional imaging methods can accurately identify intestinal strictures and can detect inflammation but not the degree of fibrosis [133]. Imaging techniques are non-invasive and allow the visualization of the entire colon to determine thickness of the bowel wall, fibrotic tissue, and any other possible complications [135]. For example, the MRI outcomes enable the investigation of the increase in intestinal wall thickness, with values between 3 and 5 mm classified as mild, >5 to 9 mm as moderate, and ≥10 mm as severe. In the meantime, imaging methods allow assessment of disease activity and follow-up on patients’ therapeutic responses [136].

## 5. In-Vivo Models of Intestinal Fibrosis

Intestinal fibrosis can be induced by several methods or chemicals. Chemically, both dextran sulfate sodium (DSS) and trinitrobenzene sulfonic acid (TNBS) can induce the fibrogenesis process. DSS at a concentration of 1.5–2% is administered to mice in drinking water in one to three repeated cycles. Firstly, animals are exposed to DSS for 7 days, and then switched to regular water for two weeks as a recovery period [137]. Another study followed the same experimental design using C57BL/6J mice, administered with 2.5% DSS in 3 repeated cycles [138]. C57BL/6J and BALB/c mice are the most appropriate strains to be used to induce fibrosis [139,140]. TNBS is usually diluted in ethanol and administered via intrarectal instillation. TNBS is thought to alter colonic proteins and induce a delayed-type hypersensitivity reaction, while ethanol disrupts the epithelial barrier. Intestinal fibrosis is induced by the repeated administration of escalating doses of TNBS over 6 weeks [141]. SAMP1/Yit mouse is a spontaneous model of intestinal fibrosis that inherently expresses inflammation and fibrosis. The knocking out of IL-10 also induces chronic inflammation and the deposition of ECM [141,142]. The injection of microbial fragments into the gut, such as the peptidoglycan polysaccharide of the bacterial wall, induces persistent bowel inflammation, resulting in fibrosis [143,144,145]. When the colon is exposed to doses of therapeutic radiation, intestinal inflammation is produced, which is eventually followed by intestinal fibrosis. Live bacterial infection through the administration of Salmonella Typhimurium via oral gavage, 24 h post treatment with streptomycin, triggers colon inflammation and the fibrogenesis process [146].

## 6. Treatment of Intestinal Fibrosis

Current treatments for IBD involve the use of 5-aminosalicylic acid, antibiotics, steroids, probiotics, and immunosuppressive agents or biologics such as monoclonal antibodies. These therapies can effectively suppress acute and chronic intestinal inflammation but cannot cease or prevent the progression into intestinal fibrosis [147,148]. On the other hand, some anti-inflammatory medications have been found to mitigate, to an extent, the stenosis of fibrosis and to have delayed surgical intervention. Currently, due to the lack of effective antifibrotic medications, the only interventional therapeutic approach for colonic fibrostenosis is still surgical resection or endoscopic balloon dilation [74,149]. In order to prevent intestinal fibrosis as a complication of IBD, it is crucial to identify new preventive drugs and strategies. Nowadays, phytochemical compounds or therapies as alternative and safer approaches are emerging to fulfill the unmet demands in the treatment of fibrosis [27,150,151,152,153]. SAMP1/Yit mouse is a spontaneous model of intestinal fibrosis that inherently expresses inflammation as well as fibrosis. The knocking out of IL-10 also induces chronic inflammation and the deposition of ECM [142].

### 6.1. Pharmacological Interventions

#### 6.1.1. Anti-Inflammatory Drugs

Aminosalicylates, corticosteroids, and antimetabolites primarily targeting inflammatory pathways have been in clinical practice for a decade. Among aminosalicylates, sulfasalazine is a prototype that has been followed by the many congeres, including mesalazine, olsalazide, and balsalazide, which constitute the first-line therapy for IBD—mainly by inhibiting proinflammatory mediators. Aminosalicylates are usually used in combination with other medications, corticosteroids or immunosuppressants because alone they cannot maintain the remission phase of the disease and delay the surgical intervention [154,155,156]. One immunosuppressive agent, azathioprine, which is a thiopurine, has been demonstrated to be effective in limiting persistent inflammation, which enables the healing of inflammatory lesions prior to the development of irreversible fibrotic tissue and intestinal wall thickening [157]. These further target immune–inflammatory cascades by mitigating inflammation and regulating the proliferation of immune cells, including T lymphocytes [158].

#### 6.1.2. Biologics

In recent years, biological agents, including the monoclonal antibodies infliximab and adalimumab, have been used to primarily target inflammatory cytokines. Additionally, integrin inhibitors, such as natalizumab and vedolizumab, have garnered attention due to their potential benefits to those who are non-responders to a conventional treatment agent. Infliximab and adalimumab are TNF-α inhibitors, while vedolizumab particularly targets the α4β7 heterodimer, which is expressed on the surface of gut-specific lymphocytes, reducing the migration of lymphocytes to the intestine. Generally, anti-TNF therapies are recommended for patients with stricture and bad prognosis as they inhibit the development of new bowel strictures. In most cases, when biologics are administrated, add-on therapy using steroids, immunomodulators or other biological agents is unnecessary.

Outcomes of several clinical studies have demonstrated that the early introduction of those medications improves patients’ quality of life, prevents the need for hospitalization and delays surgical intervention and the progression into fibrostenosis [159,160,161,162,163,164,165].

#### 6.1.3. Antifibrotic Drugs

Pirfenidone and nintedanib are FDA-approved antifibrotic drugs for the treatment of pulmonary fibrosis, but their application is limited due to various side effects [166,167]. In terms of therapeutic efficacy, they could be promising in the treatment of intestinal fibrosis, but further studies are required to investigate and validate this promise. In contrast, pirfenidone has antioxidant, anti-inflammatory, and antifibrotic actions and attenuates fibrogenesis growth factors. This inhibits the fibroblasts’ differentiation and myofibroblasts’ proliferation, which leads to the suppression of collagen synthesis and ECM deposition [168,169]. Nintedanib downregulates the fibroblasts’ growth factors, platelet-derived growth factors, vascular endothelial growth factors, and the signaling pathways responsible for the fibroblasts’ differentiation and migration [169]. In addition, it inhibits the TGF-β and the downstream genes, including collagen I and III [170]. Another study has proved that both pirfenidone and nintedanib perform antifibrotic activity by attenuating the formation of collagen-I fibrils [171].

### 6.2. Non-Pharmacological Approaches

#### 6.2.1. Surgical Resection

This is an invasive method of removing the part of the colon that has strictures. It is an effective therapeutic procedure; however, it is associated with a high rate of recurrence, approximately 70% [74]. Moreover, there is a risk of bleeding or bowel wall perforation [17,172].

#### 6.2.2. Endoscopic Stricturotomy

This is a technique that provides a safe and effective way of cutting the stricture using an endoscope and without surgical resection. This method decreases the morbidity compared with the surgical excision, but the risk of bleeding and postoperative infection is also there [17,173].

#### 6.2.3. Stenting

This approach is beneficial in preventing relapse of the strictures. However, a risk of movement or adherence of the stent to the inner mucosal layer and perforation may occur [17].

#### 6.2.4. Endoscopic Balloon Dilation

The success of dilation is measured by the ability to pass the endoscope through the stricture location. It is less invasive, maintains the bowel length intact, and limits the need for surgical resection. However, there is a risk of bowel perforation and patients may need to do the re-dilation procedure several times when new strictures develop [17,174].

## 7. Promising Phytochemicals for Future Intestinal Fibrosis Therapies

The pathogenesis and management of IBD has a link with the nutrition and dietary components. There are dietary and nutritional perspectives for the management of IBD. Considering the critical role of inflammation and immune–inflammatory cascades, in addition to oxidative stress, the focus of these perspectives is to target the associated intricate and perplexing pathways using agents of natural origin which are themselves also of value for use in dietary interventions. Many of the plants and their constituents, termed phytochemicals, have received attention due to their medicinal benefits in terms of pharmaceutical or nutraceutical development. In recent years, a convincing number of plants and phytochemicals have been evaluated in experimental models, including the in silico, in vitro, and in vivo mimicking of ulcerative colitis. The present review comprehensively presents the evidence on the therapeutic and preventive potential of phytochemicals evaluated to date in preclinical studies, as seen in the synoptic tables (Table 1) and illustrated figures presented below.

### 7.1. Asperuloside

Asperuloside (ASP) is an iridoid glycoside extracted from *Hedyotis diffusa*, a well-known folk herb in several Asian countries [175,176]. It has diverse pharmacological activities, such as anti-inflammatory, antioxidant, anticancer, and anti-obesity [177,178,179]. Asperuloside has been shown to ameliorate gut dysbiosis and regulate the gut microbiota. Additionally, it has restorative effects on the metabolic signaling in high-fat-diet-induced obesity and type 2 diabetes. It achieves these benefits by changing the gut-derived secondary metabolites and by interfering with the metabolic signaling [180]. Additionally, it has been found to mitigate ulcerative colitis in colonic tissues, indicated by a reduction in weight loss, improved disease activity index, inhibition of oxidative stress and subsequent inflammation, and maintenance of histological architecture. The benefits of ASP in IBD have been attributed to the activation of Nrf2/HO-1 signaling, which induces antioxidant responses, and the suppression of the NFκB signaling pathway [22,181,182].

It has been demonstrated that ASP can downregulate the Smad3 mRNA in IEC-6 cells with a knocked down NFκB, and can inhibit LPS-induced p-p65 levels. Studies have demonstrated that ASP prevents the transformation of the epithelial phenotype into the motile mesenchymal phenotype. In addition, the suppression of Smad3 mRNA results in the downregulation of the levels of EMT markers [179]. In the case of cancer associated with colitis, it has been shown to inhibit EMT via activation of the vitamin D receptor [183]. Moreover, ASP has been found to alleviate symptoms and inhibit tumor size by reducing α-SMA expression [179]. Other studies have also revealed that ASP has an inhibitory effect on inflammatory mediators, such as nitric oxide and prostaglandin E2, as well as other cytokines, such as IL-1, IL-6, and TNF-α [184,185]. These inhibitory actions of ASP, particularly in chronic colitis, are mediated by the downregulation of NFκB and MAPK signaling pathways [184,186]. Accordingly, the available proofs demonstrate that ASP has the potential to reduce fibrosis in the colon. However, further studies are needed to determine the effect of ASP on fibrogenesis in the intestine.

### 7.2. Berberine

Berberine is an isoquinoline alkaloid compound derived from the traditional Chinese medicine *Coptis chinensis* [187,188,189]. Research has demonstrated that berberine undergoes a wide range of pharmacological activities. Most importantly, it shows anti-inflammatory and antioxidant actions, where inflammation and oxidative stress are the primary driving factors of fibrosis. Additionally, it boosts PPARγ activities [167] and mitigates tissue fibrosis by inhibiting TGF-β1/Smad3 signaling and by downregulating α-SMA [190]. Furthermore, it has therapeutic activities for metabolic disorders, including the inhibition of blood glucose levels [191], improvement of insulin resistance [192], and reduction of hyperlipidemia [193]. In addition, it protects from mild cognitive impairment and has other antitumor [194] and immunomodulatory biological activities [195,196].

Berberine has been investigated in many studies, revealing its capability to restrain the intestinal mucosal damage caused by chronic stress and downregulate the expression of inflammatory mediators during severe abdominal infection or sepsis. Thereby, it mitigates intestinal mucosa barrier damage and minimizes intestinal wall permeability [197,198]. A rat model of UC has shown that pretreatment with berberine suppresses the levels of TNF-α, IL-1β, IL-6, IL-12, and IFN-γ, which are major proinflammatory cytokines. In addition, these findings illustrate that berberine downregulates the phosphorylation of STAT3 as well as NFκB p65, suggesting that it could effectively repress the IL-6/STAT3/NFκB pathway implicated in UC pathogenesis [198]. The main factors affecting the berberine effect are the treatment period and dose, thus extending the treatment period by more than 3 months and significantly potentiating the therapeutic effect [199]. Taken together, these results suggest that berberine could be a promising treatment for UC patients.

### 7.3. Calycosin

Calycosin (CA), is a flavonoid known as a phytoestrogen which is derived from the root of *Astragalus membranaceus* [200,201,202]. CA has anti-inflammatory, anti-oxidative stress, anti-hyperglycemic, neurological, and hepatoprotective effects [203,204,205,206,207]. Fortunately, studies have shown that CA has significant antifibrosis potential; hence, it is deemed a promising antifibrotic drug for treating organ fibrosis. CA alleviates renal and pulmonary fibrosis by limiting inflammation and oxidative stress [202,208]. Moreover, it has been reported that CA was able to successfully attenuate the severity of lung tissue damage in a fibrosis mouse model. By downregulating the AKT/GSK3β/β-catenin signaling pathway, CA suppresses TGFβ-1-induced epithelial–mesenchymal transition in alveolar epithelial cells [209]. This mechanism is also a major contributor to the development of intestinal fibrosis, by increasing collagen production and elevating the levels of extracellular matrix proteins [210].

CA has been shown to improve renal glomerulosclerosis and interstitial fibrosis in diabetes by modulating oxidative stress via IL-33/ST2 signaling [202]. CA also attenuates liver fibrosis by limiting hepatic stellate cells’ proliferation and migration, inhibiting the expression of collagen I and α-SMA in the activated hepatic stellate cells that are induced by TGF-β1. These actions are mediated by the downregulation of estrogen receptor β [211].

CA has shown some cardioprotective effects, which are mediated by PI3K/AKT pathway upregulation. It significantly downregulates the expression of α-SMA as well as the expression and deposition of collagen I and collagen III in cardiac fibrosis [21,211]. Regardless of the proven antifibrotic actions of CA, more research is required to identify the biological mechanisms underlying the protective role of CA, particularly in intestinal fibrosis.

### 7.4. Nobiletin

Nobiletin, or NOB (5,6,7,8,3′,4′-hexamethoxyflavone), is a nontoxic dietary polymethoxyflavone (PMF) that is extracted from citrus fruits [212]. It is mostly found in the peel of *Citrus sinensis* (sweet orange), *Citrus aurantum* L. (sour orange) and *Citrus paradise* (grapefruit). Furthermore, citrus fruit juice contains measurable levels of nobiletin (1–10 mg/g) [213]. NOB has lipophilic properties due to the significant presence of hydrophobic groups, which allows for high bioavailability [214]. Nobiletin has neuroprotective [215] anti-inflammatory [216,217], anti-cancer [218] and anti-oxidative properties [219]. Its anti-inflammatory effects are helpful in the treatment of IBD.

NOB and its primary metabolite, 4′-demethylnobiletin, suppress the production of inflammatory cytokines such as interleukin-1β (IL-1β), interleukin-6 (IL-6), prostaglandin E2, inducible nitric oxide synthase (iNOS) and cyclooxygenase-2 (COX-2) [193,220]. These pharmacological activities are achieved by NOB’s potential to inhibit the NFκB and ERK signaling pathways involved in the production of pro-inflammatory cytokines like TNFα [221].

In several studies, NOB has demonstrated a reduction of degranulation and pro-inflammatory mediator expression in human intestinal mast cells by functioning as an ERK inhibitor [221]. Additionally, NOB has a dual action, in alleviating both the inflammation and fibrosis associated with colitis. Firstly, it promotes the expression of the peroxisome proliferator-activated receptors (PPARγ) which serve as anti-inflammatory and antifibrotic molecules in IBD [222,223]. Secondly, it downregulates iNOS and COX-2 expression, which in turn enhances intestinal barrier function and attenuates inflammation [222,224]. On the other hand, NOB activates the IL-6/STAT3/FOXO3a signal pathway by upregulating FOXO3a phosphorylation in the cell nucleus and downregulating IL-6 and STAT3 phosphorylation, which results in induction of macrophage autophagy [225]. Furthermore, research has shown that autophagy is crucial in controlling inflammatory reactions [226,227]. NOB inhibits the inflammatory response by promoting autophagy as well as by stimulating the macrophages’ IL-6/STAT3/FOXO3a pathway [225].

### 7.5. Troxerutin

Troxerutin is a trihydroxyethylated derivative of rutin which is a natural flavonoid glycoside [228]. Troxerutin is extracted from *Saphora japonica* and is identified by its free radical antioxidant activity, which accounts for the cytoprotective effect exhibited across several cell types [229]. It has a variety of pharmacological and biological activities and shows anti-inflammatory, anticancer, antiviral, antiapoptotic, and antifibrinolytic effects [230,231,232]. Troxerutin can significantly dampen the level of oxidative stress and inflammation in the tissue of the ulcerative colon, maintaining the function of the intestinal barrier [233]. In regard to intestinal fibrosis, troxerutin administration markedly downregulates TGFβ-1 and α-SMA expression, reflecting a decrease in the formation of fibrosis. One of the research studies has proven that troxerutin is a potent candidate in the treatment of UC and intestinal fibrosis as it can relieve colonic damage and all pathological changes associated with the disease which was induced in vivo by DSS [234].

### 7.6. Olive Phenols

Major and minor components of the extra virgin olive oil are extracted by either squeezing the olives directly or by centrifuging them. More than 98% of the total weight of the oil is made up of glycerides, whereas 80% of the overall lipidic content accounts for the monounsaturated oleic acid. Over 230 chemical substances, including aliphatic and triterpenic alcohols, hydrocarbons, sterols, volatile chemicals, flavonoids, phenolic acids and antioxidants, known as polyphenols, represent the minority, with approximately 2% of the total weight of the oil [235].

These phenolic compounds modulate a number of pathways associated with inflammation, restrict the expression of pro-inflammatory molecules, and inhibit oxidative stress via counteracting the action of oxidizing enzymes and free radicals. Studies carried out both in vivo and in vitro have shown that olive oil’s polyphenols can alleviate the clinical and histological symptoms of colitis [236,237,238,239,240,241,242].

A study has been conducted recently that found that the combination of probiotics, particularly *Lactiplantibacillus plantarum*, and of fermented olives in one’s diet alleviates DSS-induced colitis by decreasing the expression of the proinflammatory cytokines, including TNF-α, IL-6, IL-1β, and PI3K signaling, and the profibrotic factors, such as TGF-β, p-Smad3, α-SMA, p-Akt, PI3K and collagens I–III. Thus, according to these outcomes, olive phenols can be used as dietary supplements in IBD to slow down disease progression and complications [243].

Hydroxytyrosol is one of the most therapeutically powerful olive phenols and has effective anti-inflammatory and antioxidant actions through the inhibition of the myeloperoxidase enzyme. This phenol decreases the expression of COX-2 and iNOS in the intestinal mucosa, mediated by the suppression of the p38 MAPK pathway [236,239]. Another study has stated that attenuating the activation of the NLRP3 inflammasome is one of the mechanisms of action of hydroxytyrosol [244]. On the other hand, other studies have found that hydroxytyrosol potentiates the NRF2 signaling pathway, which in turn promotes the activation and transcription of the antioxidants and detoxification genes [245,246].

Taken together, olive phenols are promising in terms of providing an alternative dietary and therapeutic approach to protect from IBD and its complications, most importantly fibrosis [239].

### 7.7. Total Flavones of Abelmoschus Manihot (TFA)

Total flavone of Abelmoschus (TFA) Manihot is the key flavonoid component derived from Abelmoschus Manihot. Clinical studies have found that TFA extract has anti-inflammatory, antioxidant, and gastrointestinal protection effects [247]. TFA has dual actions, as an anti-inflammatory and an antifibrotic agent. It has shown inhibition of NFκB and MAPK signaling in colitis. In addition, it suppresses the expressions of α-SMA and EMT in vivo which are induced by TGF-β1 in fibrosis. In vitro, TFA has been shown to downregulate Smad2/3 phosphorylation in the intestinal epithelial cells, which is the key trigger of the EMT and the fibrosis process [248]. A study has found that TFA can improve the microscopic and macroscopic features of fibrosis in terms of colon length and weight, stenosis, ulcerations and histological architecture. Moreover, it can diminish the disease activity index and prevent body weight loss [249]. It has been reported several times that the imbalance between MMPs and TIMPs induces the deposition of ECM, resulting in the development of fibrosis. TFA has been found to not only decrease the expression of IL-6, IL-17, TGFβ-1, collagen and α-SMA but to elevate the expression of MMP-2 and MMP-9 and downregulate that of TIMP-1, restoring the homeostasis of the ECM production and degradation [249].

**Table 1:** A summary of phytochemicals, their dose, action and mechanism of action in different experimental models. (↑—increased; ↓—decreased)

Phytochemical Compound	Experimental Model (In Vivo)	Dose and Route of Administration	Pharmacological and Molecular Mechanisms	Ref.
1. Asperuloside (ASP)	KM mice + 2.5% DSS	125 and 500 µg/kg/day, for 45 days Oral	↓ DAI, MPO, NF-κB p65, TNF-α and IL-6 ↑ Nrf2, HO-1, NQO-1, IL-10, GSH-Px, SOD	[22]
	BALB/C mice + azoxymethane (10 mg/kg, i.p.) + 2% DSS	2 mg/kg/day Oral	↓ TGF-β1/Smad3, EMT, p-p65, TNF-α and IL-1β, α-SMA, N-cadherin and vimentin ↑ IL-10 and E-cadherin	[179]
	BALB/C male mice + unilateral ureteral obstruction (UUO) model to induce renal fibrosis	14 and 28 mg/kg for 2 weeks Oral	↓ NF-κB, MAPK, TGF-β1/Smad2/3, α-SMA, collagen-III and fibronectin	[250]
2. Berberine	Wistar rats + 5% DSS to the drinking water for 7 days.	10, 30, 50 mg/kg once a day for 7 weeks Oral	↓ IL-1β, IL-6, IL-12, TNF-α and IFN-γ levels, p-STAT3, p-NF-κB p65, activation of IL-6/STAT3/NF-κB, iNOS, MPO, MDA ↑ IL-4 and IL-10	[198]
3. Calycosin	C57BL/6J mice + bleomycin administered (5 mg/kg/2 U/kg) via intratracheal instillation to induce pulmonary fibrosis	7, 14 mg/kg once a day for 3 weeks Oral	↓ Akt/GSK3β/β-catenin pathway and TGF-β1-induced epithelial–mesenchymal transition	[209]
		14 mg/kg, once a day for 21 days Oral	↓ Oxidative stress, collagen deposition ↑ Autophagy via upregulating LC3, beclin1, and PINK1 and by reducing p62. ↑ Nrf2/HO-1, SOD and expression of LAMP1 and TFEB	[208]
	BALB/C mice + cerulein (50 μg/kg) to induce acute pancreatitis	25, 50 mg/kg BW Intraperitoneal	↓ TNF-α, IL-1β, IL-6, NF-κB/p65 and phosphorylation of IκBα and p38 MAPK	[206]
	Sprague Dawley rats + STZ (30 mg/kg) i.p. + HFD for 4 weeks	5 mg/kg, for 8 weeks	↓ TGF-β, IL-33 and ST2 mRNA, NF-κB activation and pro-inflammatory cytokines	[202]
	C57BL/6J mice + methionine choline-deficient diet	2.5, 25, 50 mg/kg, once a day for 4 weeks Oral	↓ Hepatic stellate cell activation, fibrogenic gene TIMP-1 ↑ Fatty acid β-oxidation, PPARα and CPT1 protein expression	[204]
4. Nobiletin (NOB)	IL-10 knockout BALB/c mice (IL-10^−/−^), received 2% ethanol in drinking water	50 mg/kg/day in drinking water for 11 weeks	↓ TNF-α, IL-6, CCL2, collagen 3A1, intestinal wall thickness, clinical colitis, tissue damage score, rectal inflammation and bleeding score, mast cell number, and degranulation in the proximal colon.	[251]
	C57BL/6J mice, received 10% CCl4 i.p. to induce liver fibrosis	50, 100 mg/kg for 3 weeks Oral	↓ TGF-β1, α-SMA, fibronectin 1, collagen 1A1, TNF-α, IL-6, IL-1β, NLRP3, IL-18, and ROS generation ↑ Beclin1 and LC3 expression (autophagy proteins)	[252]
	C57BL/6J mice, unilateral ureteral obstructive (UUO) to induce chronic kidney injury and fibrosis	50 mg/kg, for 14 daysOral	↓ TGF-β1, fibronectin, α-SMA, collagen I, NOX4, TFR1, GPx4, SLC7A11/xCT, Bax, phosphorylated NFκB−p65, TNF-α, IL-6 mRNA and COX-2 ↑ Catalase, SOD, E-cadherin, Bcl−2, procaspase 3 and TrxR1	[253]
	Sprague–Dawley male rats—single-pass intestinal perfusion (SPIP) regions of the small intestine (i.e., duodenum, jejunum, ileum and colon)	15, 30 and 60 μg/mL at a flow rate of 0.2 mL/min.	↓ NO, iNOS, COX-2, IL-6, STAT3 and FOXO3a phosphorylation, LC3II and p62 proteins	[225]
5. Troxerutin	C57BL/6J mice, 2.5% DSS added to the drinking water for 8 days	100 and 200 mg/kg Oral	↓ Keap, NOX2, MMP-2, MMP-9, TNF-α, IL-1β, IL-17A, IL-6, IFN-γ, α-SMA, and COL3A1; the phosphorylation of JNK, Akt, p38 and ERK1/2; cleaved PARP, caspase-1, caspase-3 and Bax ↑ Nrf2, HO-1, IL-10, E-cadherin, ZO-1, Mucin-2, occludin and Bcl-2	[233]
6. Olive Phenols	C57BL/6J wild-type male mice, 2.5% DSS was added to the drinking water to induce chronic colitis for 3 cycles of 5 days each, followed by a week of normal water	10% olive cream-enriched diet	↓ IL-1β, IL-6, TNF-α, TGF-β1, p-Smad3, PI3K, p-Akt, α-SMA, and collagen I and III	[243]
	C57BL/6J mice, 3% DSS in the drinking water for 5 days	A diet enriched with extra virgin olive oil, given for 21 days	↓ TNF-α, IL-1β, COX, iNOS and p38 MAPK ↑ IL-10	[236]
7. Total flavone of Abelmoschus Manihot (TFA)	BALB/C mice, TNBS (50 mg/kg) to induce colitis via colon instillation through a catheter for 4 weeks	250 mg/kg daily for 4 weeks Intragastric instillation	↓ TGF-β, α-SMA, collagen 1A2 & 3A2, vimentin, IL-6, IL-17, TNF-α, IFN-γ, IGF-1, hydroxyproline, and TIMP-1 ↑ IL-10, MMP-2 and MMP-9	[249]
**Phytochemical Compound**	**Experimental Model** **(In Vitro)**	**Dose and Route of** **Administration**	**Findings**	**Ref.**
1. Asperuloside (ASP)	RAW264.7cells + ASP for 24 h, then stimulated with 1 μg/mL LPS for another 6 h	5, 10, 20 μM for 24 h	↓ NF-κB, TNF-α and IL-6 ↑ Nrf2/HO-1, NQO-1	[22]
	Cultured intestinal epithelial cells—6 were stimulated with 1 μg/mL LPS for 24 h, and parallelly, cells were treated with ASP	40 μM	↓ TGF-β1/Smad3, EMT, p-p65, TNF-α, IL-1β, α-SMA, N-cadherin and vimentin ↑ IL-10 and E-cadherin	[179]
2. Calycosin	CCD-18Co cells were treated with CA for 24 h after stimulation with TGF-β1	12.5, 25, 50, 100, 200, 400, 800 μM	↓ TGF-β1/Smad signaling pathway ↓ mRNA expression levels of TGF-β1, Smad2, -3, -4, α-SMA and collagen I↑ Smad7	[13]
3. Troxerutin	H9C2 cells were transfected with HIF-1α-siRNA (50 nM), 48 h later, cells were incubated with 10 µM troxerutin for 1 h	10 µM for 1 h	↓ PI3K/Akt/HIF-1α, NO, iNOS, COX-2, IL-6, STAT3 and FOXO3a phosphorylation, LC3II and p62 proteins	[228]
4. Total flavone of Abelmoschus Manihot (TFA)	Rats’ intestinal epithelial cells (IEC-6), induced with 10 ng/mL TGF-β1 for 48 hr	0, 5, 10 and 15 μg/mL	↓ Mesenchymal proteins (vimentin and N-cadherin), TGF-β1-induced EMT, and migration of the cells; p-Smad2/3, p38, JNK and ERK1/2 and their phosphorylated forms. ↑ Epithelial markers (E-cadherin and ZO-1)	[248]

## 8. Herbal Extracts

In addition to phytochemicals, numerous plant extracts have been evaluated in different experimental models of IBD. These are presented in the tables (Table 2) and are elaborated on further.

### 8.1. Boswellia and Scutellaria Extracts

*Boswellia serrata* is known for its gum resin, which has been used to treat inflammatory and arthritic conditions for a long time [254]. The main active constituents of Boswellia extracts are the boswellic acids (BA) which belong to ursane, a type of pentacyclic triterpene. It has been reported that the gum resin extracts of Boswellia exhibit immunomodulatory and anti-inflammatory properties [255,256]. The therapeutic effect of BA in attenuating tissue injury and the associated inflammatory responses facilitates its antifibrotic activity [257].

BA has shown an inhibition of TNF-α and the degree of arachidonate 5-lipoxygenase (5-LOX) enzyme activity. Leukotrienes (LT) are significant mediators of inflammation and 5-LOX has been identified as a target for BA. Therefore, the inhibition of LT synthesis by BA may be the underlying mechanism of BA’s anti-inflammatory actions [258,259]. Additionally, BA could significantly dampen the TGF-β1-induced fibrosis, where it has been reported that BA may directly exert its antifibrotic effect by the downregulation of TGF-β1 [260,261,262,263].

*Scutellaria baicalensis* Georg and its dried root, *Scutellariae radix*, are traditional herbs that have been used as antioxidants and anti-inflammatories, particularly in the treatment of gastroenteritis [264]. Baicalein, baicalin, wogonin, wogonoside, oroxyloside and the methanolic extract of *Scutellariae radix* are the major active flavonoid components of the *Scutellaria baicalensis* and these prevent the accumulation of collagen and display potent antifibrotic activities [264,265,266,267]. This herb quenches inflammation through the PI3K/Akt/NF-κB pathway and inhibits both TGF-β, the main driver of fibrosis, and TIMPs, which are responsible for degrading the excessive ECM to maintain the balance between ECM formation and degradation; hence, protecting from the progression into fibrosis [268,269].

### 8.2. Gentianopsis paludosa

*Gentianopsis paludosa* is an annually growing herb in the family of *Gentianaceae gentianopsis* [270]. Among traditional medicine, this entire plant is used to cure gastroenteritis, nephritis, hepatitis, conjunctivitis, dyspepsia, fever, influenza, and bloody diarrhea, which is one of the main features of fibrosis associated with UC [271,272]. It has been reported that *Gentianopsis paludosa* could significantly downregulate the expression of α-SMA, collagen I, and collagen III, which are overexpressed in UC-associated fibrosis. On the other hand, it can replenish the levels of E-cadherin, which is crucial for the maintenance of gut homeostasis and the integrity of the epithelial lining and intestinal wall barrier [273,274].

### 8.3. Flavonoid-Rich Citrus Extracts

Citrus flavonoids (CFs) are a class of dietary flavonoids that includes diverse polyphenolic compounds obtained from the citrus plants [275]. The pure total flavonoids of citrus (PTFC) have been extracted and purified from the dry, ripe peels of the citrus species. In citrus plants, over 80 natural flavonoids have been determined, with the major identified flavonoids being nobiletin, naringin, neohesperidin, narirutin, and hesperidin, which together compose the PTFC [276,277]. CFs have received attention recently due to their antioxidant and anti-inflammatory attributes [278]. They promote the inhibition of oxidative stress and inflammation in the gut lumen, regulate the intestinal barrier permeability and the favorable alteration of the gut microbiota, and immunomodulation [279]. For example, nobiletin has been shown to protect against body weight loss and damage of the intestinal barrier permeability and to decrease disease activity index score after induction of colitis with barrier disruption in mice and rats by DSS and TNBS, respectively. Additionally, naringenin has been found to prevent body weight loss and a shortening of the colon length. Furthermore, it suppresses the proinflammatory cytokines and oxidative stress in mice colitis models [280].

### 8.4. Cinnamon Extract (CE)

Cinnamon extract (CE) is extracted from the cinnamon bark of *Cinnamomum ceylanicum*. It exhibits antioxidative, anti-inflammatory, anti-allergic, antineoplastic, and antidiabetic actions [281,282,283,284,285]. Cinnamaldehyde (CA) is the major active constituent of CE and it inhibits the proinflammatory mediators and cytokines when taken orally as a treatment. CE and its active ingredient, CA, have shown a notable suppression in the activation and phosphorylation of NF-κB and downregulation of the proinflammatory cytokines, including IL-6, IL-1β, CCL2, and chemokine (C-X-C motif) ligand- 8 (CXCL8), stimulated by LPS in the fibroblasts.

Regarding fibrosis, a study has illustrated that the expression of MMP-1 is upregulated in the colon of IBD patients; however, with the administration of CE, the levels of MMP 3, 9, and 13 were suppressed, resulting in a decrease of collagen-I production and ECM deposition. Accordingly, this suggests that CE has the potential to be an antifibrotic [153].

**Table 2:** A summary of plant extracts, their dose, duration, action and molecular mechanisms in different experimental models. Abbreviations: connective tissue growth factor—CTGF, alpha-smooth muscle actin—α-SMA, aspartate transaminase—AST, alanine aminotransferase—ALT, interferon gamma—IFNγ, chemokine (C-X-C motif) ligand-8—CXCL8, C-C motif ligand-2—CCL2, chronic obstructive pulmonary disease—COPD (↑—increased; ↓—decreased).

	Experimental Model (In Vivo)	Dose and Route of Administration	Findings	Ref.
Boswellia and Scutellaria	Boswellia and Scutellaria: Sprague–Dawley rats + TNBS-induced colitis/fibrosis given by intrarectal instillation (15 mg/mL)	50 (Boswellia) and 150 (Scutellaria) mg/kg/day Oral	↓ TGF-1β/Smad3 pathway, α-SMA, collagen types I-III and CTGF ↑ Smad7	[249]
	Boswellia: Swiss albino rats exposed to γ irradiation (IR). Bleomycin (BL) was injected (0.15 U in 25 μL 0.9% normal saline) to induce lung fibrosis	1 g/kg body weight/day dissolved in distilled water for 21 days after 7 days of BL induction of lung fibrosis	↓ TGFβ-1, TNF-α, 5-hydroxyproline, 5-lipoxygenase enzyme, fibrotic lesions, and inflammatory cells ↑ Glutathione, SOD, and catalase.	[263]
	Scutellaria: (A) Sprague–Dawley rats + bile duct ligation or by oral CCl4 (1 mg/kg) which was given twice a week for 28 days (B) Sprague–Dawley rats + COPD, induced by exposure to tobacco smoke	Methanol extract of Scutellaria 150 mg/kg once a day orally by gavage for 28 days 1.5, 3, 6 mg/kg/day, for 6 daysIntragastric	↓ AST, ALT, hydroxyproline (↓ collagen accumulation), expression of α-SMA and malondialdehyde (MDA) (↓ lipid peroxidation) ↓ TNF-α, IL-6, IL-8, TGF-β1, MMP-2, MMP-9, TIMP-1, p-AKT and p-NF-κB ↑ IL-10	[264,269]
*Gentianopsis* *paludosa*	Wistar rats + TNBS (150 mg/kg) to induce intestinal fibrosis	11.2, 27.0, 89.0, 119.2, 140 mg/kg for 28 days Intragastric	↓ α-SMA, collagen I & III ↑ E-cadherin	[274]
Flavonoids-rich citrus extracts	Nobiletin: C57BL/6J mice + 3% DSS	Given in diet, 0.01% or 0.25 mmol in 1 kg of diet wt/wt) for 1 week	↓ Colon shortening, body weight loss, and DAI score. ↑ Claudin-7	[286]
	Naringenin: BALB/c mice + 2% DSS	Given in diet, 0.3% or 0.3 g/100 g of diet wt/wt for 9 days	↓ IFNγ, IL-6, IL-17A, MIP-2, body weight loss, and colon shortening. ↑ intestinal TJ barrier protection	[287]
Cinnamon extract (CE)	In vivo: IL-10^−/−^ Balbc/J In vitro: Patients’ intestinal fibroblasts were cultured in a media containing CE or CA	In vivo: 4.5 mL/kg/day of CE prepared in 70% ethanol and added to the drinking water for 11 weeks. In vitro: 0.1–10 μL/mL overnight	↓ Collagen deposition, MMP, p-NFκB, IL-6, CXCL8, and CCL2	[153]

## 9. Traditional Herbal Medicine

In addition to phytochemicals, and plant extracts, various plant-based agents popular in traditional Chinese and Indian medicines have been evaluated in different experimental models of IBD. These are presented in the tables (Table 3) and elaborated further.

### 9.1. Ankaferd

Ankaferd blood stopper (ABS) is a hemostatic compound which was originally used in Turkish conventional medicine [288]. It is a distinctive medicinal product composed of a mixture of various plant extracts isolated from *Thymus vulgaris* (5 mg/100 mL), *Urtica dioica* (6 mg/100 mL), *Alpinia officinarum* (7 mg/100 mL), *Vitis vinifera* (8 mg/100 mL) and *Glycyrrhiza glabra* (9 mg/100 mL) [289]. Studies have shown that ABS has anti-inflammatory actions, modulating the inflammatory response through effects on the endothelium, angiogenesis, and cytokines [290]. The major mechanism underlying this action of ABS is the formation of an encapsulated protein network and the increase of the aggregation of erythrocytes [291].

Besides the hemostatic effect, a previously conducted study has observed that ABS has an antimicrobial effect against different pathogens [292]. Although the anti-infective activity of ABS remains to be elucidated, it may be associated with its hemostatic functions, which target the protease-activated receptor-1 (PAR-1), endothelial protein C receptor (EPCR), and plasminogen activator inhibitor-1 (PAI-1), affecting coagulation as well as vascular endothelium [293,294].

Moreover, ABS induces the mediators associated with wound healing to increase vascular and cellular proliferation via the reduction of tissue necrosis [295,296,297]. Colitis treated with ABS exhibits lower microscopic and macroscopic scores of colonic inflammations, with an enhancement of mucosal healing upon administration of a sufficient dose [293]. To this end, further studies are required to discover the broad anti-inflammatory actions of ABS.

### 9.2. Daikenchuto (DKT)

Daikenchuto is a traditional Japanese and Chinese herbal medicine frequently prescribed for the relief of intestinal inflammation, and constipation, and to improve post-intestinal surgery, adhesion, and gastrointestinal motility. It is made up of several crude substances including ginger (*Zingiberis rhizoma*), ginseng (*Panax ginseng*), dried Japanese pepper or jalapeno pepper (*Zanthoxyli fructus*), and malt sugar [298,299].

DKT dramatically diminishes mucosal damage, inflammatory adhesions of the colon, and the levels of the pro-inflammatory cytokines including TNF-α and IFN-γ [298]. DKT undertakes its antifibrotic actions via activating the transient receptor potential ankyrin 1 (TRPA1) of intestinal myofibroblasts resulting in the downregulation of the fibrotic pathway induced by TGF-β1 and other fibrosis factors, such as collagen-1A1 and α-SMA. Additionally, the upregulation of myofibroblasts’ TRPA1 by DKT is associated with the negative regulation of collagen synthesis [299,300,301,302].

### 9.3. Danhong Injection (DHI)

Danhong injection (DHI) is a traditional medicine extracted from *Carthami tinctorii* Flos and *Salviae miltiorrhizae* Radix. Salvianic acid A, salvianic acid B, rosmarinic acid, and protocatechuic aldehyde are the major active constituents of DHI [303]. One study has found that DHI has anti-inflammatory and antioxidant effects [304,305]. Another subsequent study has demonstrated that DHI could protect from postoperative intestinal adhesion by attenuating inflammation, oxidative stress, and collagen accumulation by inhibiting α-SMA and fibrin networking and promoting fibrinogenesis [306]. The mechanism behind its therapeutic actions results from its capability to increase MMP-9 and tissue-type plasminogen activator (t-PA) levels. Both enzymes act to maintain the balance between ECM synthesis and degradation, preventing excessive ECM deposition and fibrinogenesis [306,307].

### 9.4. Huangqi Decoction

Huang-lian-Jie-du Decoction (HQD) is a widely described traditional medicine that is well known for its anti-inflammatory and antioxidant effects, allowing its application in the treatment of UC [308]. HQD consists of a 3:3:2:2 ratio of Coptidis Rhizoma, Gardeniae Fructus, Scutellariae Radix, and Phellodendri Chinensis Cortex, respectively [309].

HQD performs its anti-inflammatory and antioxidant actions by modulation of the PPARγ and inhibition of NF-κB signaling pathways [310]. In a liver fibrosis study induced in rats, HQD has been shown to significantly alleviate fibrosis by suppressing the expression of TGF-β1; hence, inhibiting myofibroblast activation and proliferation. These findings suggest that HQD might be an effective antifibrotic agent by targeting the TGF-β1/Smad3 and the ERK1/2 signaling pathways [311]. Additionally, many other studies have confirmed that HQD is effective at relieving liver fibrosis via inhibition of fibrogenesis proteins and downregulation of both the TGF-β/Smad and Wnt/β-catenin pathways [312,313,314,315]. Another recent study has revealed that HQD could inhibit the activation and proliferation of the hepatic stellate cells driven by TGF-β1 in hepatic fibrosis, together with the downregulation of the expression of α-SMA and collagen-1A2. Afterwards, the authors concluded that HQD modulates the long noncoding RNA-C18orf26-1/miR-663a/TGF-β1/TGF-βRI/p-Smad2 pathway to achieve these activities [316].

Similarly, in the case of renal fibrosis, HQD could ameliorate the ipsilateral kidney fibrosis in a dose-dependent manner by downregulation of TGF-β1, TGF-β receptor I and II, Smad2, P-Smad2, Smad4, α-SMA, and collagen I, III, and IV expression. However, it was able to upregulate that of Smad7 [313].

**Table 3:** A summary of traditional herbal medicines, dose, duration, action and molecular mechanisms in different experimental models. Abbreviations: nitric oxide—NO, heat shock protein-47—HSP47, nuclear factor erythroid 2-related factor 2—Nrf2, Kelch-like ECH-associated protein-1—Keap1. (↑—increased; ↓—decreased).

Traditional Herbal Medicine	Experimental Model (In Vivo)	Dose & Route of Administration	Findings	Ref.
1. Ankaferd blood stopper (ABS) (Turkish/Asian herbal medicine)	Wistar albino rats + 2 mL 4% acetic acid to induce colitis	2 mL/day, for a week Rectal injection	↓ MDA, NO in the colonic tissue ↑ SOD	[293]
	Wistar albino rats + end-to-end colonic anastomosis	0.1 mL Topical (wiped on the anastomosis line)	↑ colon anastomosis healing by ↑ collagen formation and neovascularization	[317]
2. Daikenchuto (Japanese/Chinese herbal medicine)	Wistar rats + 0.25 mL of TNBS (120 mg/mL) dissolved in 50% ethanol delivered to the colon lumen for a week	900 mg/kg/day for a week, composed of (20 mg/kg) of Japanese pepper, (50 mg/kg) of processed ginger, (30 mg/kg) of ginseng radix, and (800 mg/kg) of maltose powder Gastric intubation	↓ TGF-β1, collagen-I, α-SMA, and intestinal HSP47	[298]
	Mice + TNBS, prepared in 30% ethanol/PBS (10 mg/mL; 50 μL), was delivered weekly for 6 weeks	5 mg/kg/day for a week Enema	↓ TGF-β1, α-SMA, collagen-I, Smad2/3, p-Smad2, and p38-MAPK ↑ mRNA and protein expression levels of transient receptor potential ankyrin 1 (TRPA1) channel in myofibroblasts	[299]
3. Danhong injection (DHI) (Chinese herbal medicine)	Sprague–Dawley (SD) rats + cecal abrasion surgery	0.8 mL of 3 different doses of DHI (1 mL/kg, 2 mL/kg and 4 mL/kg) DHI was injected I.V daily in the tail for a week	↓ TNF-α, TGF-β1, α-SMA and plasminogen-activating inhibitor (PAI), NF-κB phosphorylation, ROS, ↑ MMP-9, Nrf2, and tissue-type plasminogen activator (t-PA) in the adhesion tissues.	[306]
4. Huangqi decoction (Chinese herbal medicine)	Sprague–Dawley rats + bile duct ligation (BDL) to induce liver fibrosis	17.276 mg/100 g for 4 weeks Oral	↓ Albumin, ALT, AST, TGF-β1, α-SMA, collagen in tissue, Smad3, ERK1/2, p-Smad3 and p-ERK1/2	[311]
	BABL/C mice + 3.5% DSS in the drinking water for 7 days	9.2, 4.6, 2.3 g/kg Oral	↓ TNF-α, IL-1β, MPO, NO, MDA, and NF-κB p65. ↑ IL-10, Nrf2/Keap1, GSH, ZO-1, SOD and occludin	[308]
	Hepatic stellate cells	0, 5, 10, 25, 50 and 100 mg/mL for 24–72 h.	↓ Col 1A2, α-SMA, p-Smad2, TGFβ-RI, activation and proliferation of hepatic stellate cells induced by TGFβ-1 by ↑ expression of miR-663a and ↓ expression of noncoding RNA-C18orf26-1	[316]

## 10. Discussion

Intestinal fibrosis is one of the end-stage complications of IBD. It is characterized by recurrent intestinal tissue injury, due to chronic inflammation, accompanied by failure of repair, resulting in excessive production and accumulation of ECM-promoting fibrosis with the formation of strictures and narrowing of the intestinal lumen [318,319].

The key target in the treatment of fibrotic IBD is the inhibition of the chronic inflammation that drives the development of stenosis and fibrosis [320]. Despite the noteworthy advances in the management of IBD, no apparent decrease in the prevalence of intestinal fibrosis has been noticed [321]. Diverse classes of therapy, with different modes of action, have been developed to diminish the risk of fibrosis, including anti-adhesion agents, sphingosine-1-phosphate modulators and inhibitors of the IL-12/23 pathways, the IL-36, JAK/STAT signaling pathway, the TNF-like ligand 1A (TL1A) and phosphodiesterases (PDEs) [322]. Currently, no drug has been approved as a particular intestinal antifibrotic agent. In the clinical settings, anti-inflammatory medications, such as corticosteroids and mesalazine, immunosuppressive drugs, such as methotrexate and azathioprine, and biologics are considered for the control of fibrostenosis as inflammation is the primary cause of fibrosis [320,321]. However, approximately 80% of IBD patients with strictures are refractory to those medications, so they eventually undergo endoscopic balloon dilation or surgery [323]. Furthermore, myelosuppression, infections, liver toxicity, hypersensitivity reactions, and malignancy are among the adverse effects of these drugs. On the other hand, biologics, particularly anti-TNFα drugs, are associated with infusion reactions and a gradual loss of response due to the formation of anti-drug antibodies [324,325]. Thus, the emergence of other alternative treatment options is necessary [321].

**Figure 5:**
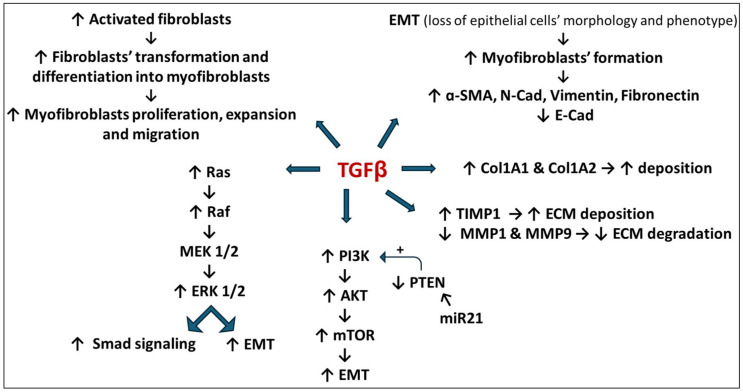
An overview of role of TGFβ in the pathogenesis of intestinal fibrosis. Additionally, ASP, Nobiletin, Olive phenols, TFA, and Huangqi decoction have an inhibitory effect on the MAPK pathway. Abbreviations: Rat sarcoma-small GTP-ase—Ras, Raf-1 proto-oncogene—Raf, mitogen-activated protein kinase kinase-1/2—MEK, extracellular signal-regulated kinases—ERK, phosphoinositide 3-kinase—PI3K, mammalian target of rapamycin—mTOR, epithelial–mesenchymal transition—EMT, phosphatase and tensin homolog—PTEN, tissue inhibitors metalloproteinases—TIMP, matrix metalloproteinases—MMP, collagen typeI alpha1/2—Col1A1/2 (↑—increased; ↓—decreased).

TGF-β is the master driver of intestinal fibrosis (Figure 5) by activating either Smad-dependent or Smad-independent signaling pathways. In the Smad-dependent pathway, Smad2 and 3 are immediately phosphorylated by the TGF-β receptor complex, which then combines with Smad4, producing a complex that crosses the nucleus and induces the transcription of the fibrosis genes, EMT process, and ECM deposition. Smad6 and Smad7 can negatively regulate this stimulatory signaling by hindering the Smad2/3 phosphorylation and promoting the degradation of the TGF-β receptor. On the other hand, the NF-κβ, MAPK, and PI3K pathways, EMT, and the fibroblasts’ phenotype transformation into myofibroblasts are activated by TGF-β to induce fibrosis, independent of the Smad pathway [326]. Additionally, the elevated pro-inflammatory cytokine levels (TNF-α, IL-6, IL-1β, IL-17, and interferon gamma -(IFNγ)) accompanied by the chronic inflammation contribute to the induction of the NF-κβ and MAPK pathways, myofibroblasts, collagen synthesis, and MMPs and TIMPs imbalance [78].

The TGF-β/Smad pathway and the downstream fibrostenosis can be targeted and attenuated by ASP, Calycosin, Troxerutin, Olive phenols, TFA, Boswellia and Scutellaria extracts, Daikenchuto and Huangqi decoction. In particular, α-SMA and ECM deposition, including collagen I and III, can be inhibited by *Gentianopsis paludosa*, CE, Daikenchuto, Danhong injection, and Huangqi decoction. Regarding cytokines, ASP, Berberine, Nobiletin, Olive phenols, TFA, Boswellia and Scutellaria extracts, CE, Ankaferd, and Daikenchuto can markedly suppress their expression. ASP, Berberine, Nobiletin, TFA, Boswellia and Scutellaria extracts, CE, and Huangqi decoction negatively regulate NF-κβ signaling.

While Calycosin, Troxerutin, Olive phenols, and Scutellaria extracts can impede the PI3K/Akt pathway, ASP, Calycosin, Troxerutin, Olive phenols, and Danhong injection modulate the Nrf2/HO-1 signaling, which is essential in quenching oxidative stress and allowing for the tissue repair that is mediated by promoting the synthesis of antioxidant genes [327]. In addition, Nobiletin enhances the expression of PPAR-γ which has potent anti-inflammatory and antifibrotic effects [223]. See Table 4 for all compounds and their targets.

The conductance of further clinical trials and the emergence of more drugs are imperative with the increase in the number of cases worldwide [322]. We have shed light on the clinical research trials registered in the ClinicalTrials.gov database over the last 5 years, including the inactive, ongoing and completed studies, as well as studies of unknown status. Most of the studies have focused on the diagnosis and the identification of biomarkers implicated in the disease which, in turn, and from their perspective, push further research towards the discovery of novel therapeutic targets and drugs. One of the studies hypothesized that intestinal fibrosis can be triggered independently of inflammation by microbiota dysbiosis. With respect to diagnosis, MRI, and PET using radioactive tracers that inhibit fibroblast activation proteins are suggested to be the most advanced and secure non-invasive techniques with which to diagnose intestinal fibrosis. One study has postulated that sirolimus, also known as rapamycin, a macrocyclic antibiotic with immunosuppressive and antineoplastic characteristics, might serve as a promising rescue drug for refractory CD patients with strictures or stenosis.

To this end, clinical studies on intestinal fibrosis therapy are notably insufficient. In addition to the adverse effects and resistance to the standard drugs, all of the studies note that the identification of other therapeutic options is inevitable. In this context, research studies on phytochemicals displaying antioxidant, anti-inflammatory, and anti-fibrotic properties may address the lack of effective treatments and potentially provide an alternative therapeutic approach to the development of effective and safe intestinal fibrosis drugs that can be administered as adjunctive therapy to existing medications so as to synergize their actions and overcome the loss of response. For clinical practice, still further in-depth studies about phytochemical drug candidates are essential to clinically validate safety and efficacy, standardize effective therapeutic doses, and enhance bioavailability and pharmaceutical formulations. Meanwhile, reliable biomarkers are required to diagnose and detect patients’ responses to treatment. On a cautionary note, while phytochemicals are often regarded as safer alternatives to conventional treatments due to their natural origins, herb-induced liver injury (HILI) remains a significant concern, particularly when exploring their therapeutic potential in conditions such as intestinal fibrosis. The liver, as the primary site for metabolizing these compounds, is especially susceptible to toxicity, especially when the mechanisms of action or interactions with other medications are not fully understood. A recent systematic review and meta-analysis provides a detailed discussion of the various phytochemicals linked to HILI [328]. In the context of intestinal fibrosis, the anti-inflammatory and antioxidant properties of some phytochemicals hold promise. However, the risk of HILI must be carefully monitored to ensure that the benefits of using herbal remedies do not outweigh the potential for liver damage.

## 11. Conclusions

In conclusion, intestinal fibrosis is a common and severe complication of inflammatory bowel disease (IBD), characterized by excessive fibrous tissue accumulation that leads to bowel obstruction, strictures, and often necessitates surgical intervention. Despite advances in understanding the complex pathogenesis of fibrosis, which involves fibroblast activation, immune cell infiltration, and dysregulation of signaling pathways such as TGF-β and Wnt, no antifibrotic therapies have been approved, leaving patients with limited treatment options that focus on symptom management rather than halting disease progression. The exploration of phytochemicals, bioactive compounds derived from plants, has emerged as a promising avenue for addressing this therapeutic gap. These compounds have demonstrated potential in modulating fibrosis-related pathways, such as inflammation and oxidative stress, with potentially fewer side effects than synthetic drugs. Although still in early research stages, phytochemical-based therapies hold promise for more effective and safer treatment options, and further research to validate their efficacy in clinical settings could lead to innovative approaches for managing intestinal fibrosis in IBD patients.

## 12. Future Perspectives

The future perspectives highlighted in this review on phytochemicals as potential treatments for intestinal fibrosis in inflammatory bowel disease (IBD) reveal several promising avenues. Phytochemicals, like Asperuloside, Berberine, and Calycosin, have demonstrated anti-inflammatory, antioxidant, and antifibrotic effects in preclinical models, offering potential alternatives or supplements to traditional therapies. Moving forward, research should focus on translating these findings into clinical trials to evaluate the safety, efficacy, and therapeutic potential of these compounds in human IBD patients. Additionally, a deeper understanding of the molecular mechanisms by which these phytochemicals influence fibrosis pathways, such as the TGF-β/Smad and NFκB signaling, could uncover new therapeutic targets. Integrating phytochemicals into the management of fibrosis may help address the unmet clinical need for effective antifibrotic treatments.

## Figures and Tables

**Table 4:** Overview of molecular targets modulated by phytochemicals. (↑—increased; ↓—decreased)

↓ TGF-β/Smad	↓ NFκB/Cytokines	↓ MARK	↓ PI3K/Akt	↓ β-Catenin	↓ Collagen	↓ Vimentin
1. Asperuloside 2. Calycosin 3. Troxerutin 4. Nobiletin 5. Olive phenols 6. Total flavone of Abelmoschus Manihot 7. Boswellia and Scutellaria extracts 8. Daikenchuto 9. Huangqi decoction	1. Asperuloside 2. Berberine 3. Calycosin 4. Nobiletin 5. Troxerutin 6. Olive phenols 7. Total flavone of Abelmoschus Manihot 8. Boswellia and Scutellaria extracts 9. Flavonoid-rich citrus extracts 10. Cinnamon extract	1. Asperuloside 2. Calycosin 3. Troxerutin 4. Olive phenols 5. Total flavone of Abelmoschus Manihot	1. Calycosin 2. Troxerutin 3. Olive phenols	1. Calycosin	1. Asperuloside 2. Calycosin 3. Nobiletin 4. Troxerutin 5. Olive phenols 6. Total flavone of Abelmoschus Manihot 7. Boswellia and Scutellaria extracts 8. Gentianopsis paludosa 9. Cinnamon extract 10. Daikenchuto	1. Asperuloside 2. Total flavone of Abelmoschus Manihot
**↓ N-Cadherin**	**↑ E-Cadherin**	**↑ Smad7**	**↓ TIMP**	**↑ MMP**	**↑ IL-10**	**↑ Nrf2/HO-1**
1. Asperuloside 2. Total flavone of Abelmoschus Manihot	1. Asperuloside 2. Total flavone of Abelmoschus Manihot 3. Troxerutin 4. Nobiletin 5. Gentianopsis paludosa	1. Calycosin 2. Boswellia and Scutellaria extracts	1. Calycosin 2. Total flavone of Abelmoschus Manihot	1. Asperuloside	1. Asperuloside 2. Berberine 3. Troxerutin 4. Olive phenols 5. Total flavone of Abelmoschus Manihot 6. Boswellia and Scutellaria extracts	1. Asperuloside 2. Calycosin 3. Troxerutin
